# Failure-to-rescue in surgical practice: a systematic review and critical appraisal of recent clinical studies

**DOI:** 10.3389/fsurg.2026.1827601

**Published:** 2026-05-19

**Authors:** Claudia Varrella, Carlo Benzoni, Alberto Biondi, Domenico D'Ugo, Laura Lorenzon

**Affiliations:** 1 Fondazione Policlinico Universitario Agostino Gemelli IRCCS, Catholic University of the Sacred Heart, Rome, Italy; 2 Quality Improvement Italia, Rome, Italy; 3 Catholic University of the Sacred Heart, Rome, Italy

**Keywords:** failure to rescue, healthcare quality, patient safety, postoperative complications, quality indicators, surgical outcomes, systematic review

## Abstract

**Background:**

Failure to rescue (FTR)—the inability to prevent mortality following a complication—is a critical quality and safety metric reflecting a healthcare system's capacity to recognize and respond to patient deterioration. Despite its conceptual prominence, the actual use of FTR in surgical research remains poorly characterized. This systematic review addresses the gap by examining how FTR has been operationalized in recent clinical surgical studies, which subspecialties and regions contribute to the evidence base, and what bibliometric patterns reveal about knowledge uptake.

**Methods:**

A systematic literature search was conducted following PRISMA guidelines for clinical studies on FTR published between 2019 and 2024. PubMed and PubMed Central were searched using structured queries. Studies were assessed for risk of bias using the Newcastle-Ottawa Scale. Primary outcomes were: (1) temporal trends in FTR publications, (2) FTR's use as primary or secondary outcome, and (3) its operationalization as an indicator of structure, process, or clinical outcome according to Donabedian's framework. Secondary outcomes included geographic distribution of contributing authors, surgical subspecialty representation, and bibliometric impact (Scopus and Google Scholar citations, Altmetric scores).

**Results:**

Of 322 articles screened, 38 met inclusion criteria. The literature consisted predominantly of multicenter retrospective studies (median sample size 29,482 patients), with FTR serving as the primary outcome in 71% of studies. FTR was most frequently operationalized to assess hospital structure (44.8%), followed by process (28.9%) and clinical outcome (26.3%). Publication output showed moderate growth from 2019 to 2021 (9–11 manuscripts/year) but declined thereafter. High-income countries dominated contributions, with the USA accounting for 47.4% of studies and Europe for 42.1%. Abdominal surgery and related subspecialties (emergency, hepatobiliary, colorectal) represented 58% of publications; emergency surgery and trauma contributed 18.4%. Despite recent publication dates, studies achieved substantial bibliometric impact: mean citations were 18.4 (Scopus) and 23.2 (Google Scholar), with 76% published in first-quartile surgical journals (mean impact factor 6.2). No significant differences in bibliometric metrics were observed across indicator categories (structure/process/outcome; *p* > 0.60) or surgical subspecialties (*p* > 0.62), suggesting a homogeneous field in terms of impact regardless of application.

**Conclusions:**

Despite growing recognition of FTR as a quality metric, the volume of high-quality clinical surgical studies remains limited, geographically concentrated, and subspecialty-skewed. The field is characterized by high impact but narrow scope: predominantly retrospective North American and European studies in abdominal and emergency surgery. Standardization of FTR definitions, expansion to low- and middle-income countries, broader subspecialty engagement, and prospective interventional studies are urgently needed to advance FTR from a surveillance metric to an actionable quality-improvement tool. The field must transition FTR from a passive monitoring metric to an active driver of quality improvement to realize its potential to save lives and reduce preventable postoperative mortality.

**Systematic Review Registration:**

INPLASY, identifier 202630097.

## Introduction

Failure to rescue (FTR) is defined as the inability to prevent mortality following a postoperative complication. Unlike conventional mortality metrics, FTR isolates the system's effectiveness in recognizing deterioration and escalating care, making it a powerful indicator of healthcare quality and safety (1). FTR was introduced in 1992 by Silber and colleagues to explain mortality disparities between hospitals with similar complication rates: hospitals with higher mortality did not necessarily have more complications but were less capable of rescuing patients once complications occurred (2).

FTR is calculated as the ratio of postoperative deaths (numerator) to the number of patients experiencing postoperative complications (denominator) (1, 2). This metric reflects three interdependent domains of Donabedian's quality framework (3): *structure* (resources, staffing, volume), *process* (recognition, escalation protocols, timely intervention), and *clinical outcome* (differential mortality rates). By bridging these domains, FTR offers a system-level assessment of perioperative care pathways.

Despite its conceptual importance, measuring FTR remains challenging. A recent systematic review identified 131 distinct complications used to define FTR across 295 studies, with a median of 10 complications per study and substantial variability in inclusion criteria (4). This heterogeneity reduces generalizability and complicates cross-institutional and international comparisons (5, 6). Furthermore, the adoption of FTR is fragmented: not all surgical subspecialties routinely report FTR, and the literature remains concentrated in high-income countries, raising questions about global representativeness and applicability (7, 8).

Recent work has highlighted both the promise and limitations of FTR. Studies in cardiac surgery, abdominal surgery, and emergency general surgery have demonstrated associations between FTR and hospital volume, nurse staffing ratios, rapid response teams, and time to intervention (9–11). However, many of these studies are retrospective, single-country analyses with limited ability to inform actionable quality-improvement interventions (12). There is a need for a comprehensive appraisal of how FTR is currently used in surgical research, which populations and settings are studied, and how the field is evolving.

Unlike prior conceptual reviews or those focused on broader in-hospital deterioration (13), this systematic review specifically examines *clinical surgical studies* published in the most recent five-year period (2019–2024). Our objectives are threefold:

1. **Operationalization**: To assess how FTR is used as an outcome (primary vs. secondary) and as an indicator (structure, process, or clinical outcome) in contemporary surgical research.
2. **Geographic and subspecialty distribution**: To map which countries and surgical subspecialties contribute to FTR evidence and identify underrepresented areas.
3. **Bibliometric impact**: To quantify the scholarly attention and knowledge uptake of FTR studies through citation metrics and Altmetrics, and to examine whether impact varies by indicator type or subspecialty.

By synthesizing these dimensions, we aim to identify critical gaps in the current FTR literature and propose a roadmap for future research that broadens the metric's reach and enhances its utility for quality improvement.

## Methods

### Study design and protocol

This systematic review was registered in **INPLASY** [Registration ID: (202630097)] and conducted in accordance with the Preferred Reporting Items for Systematic Reviews and Meta-Analyses (PRISMA 2020) guidelines (14). The protocol was registered retrospectively after study completion but before manuscript submission.

### Eligibility criteria: PICOS framework

Studies were selected according to the following pre-specified PICOS criteria:

- Population: Adult patients (age ≥18 years) undergoing any surgical procedure in any surgical subspecialty (general surgery, emergency surgery, cardiovascular surgery, thoracic surgery, hepatobiliary surgery, colorectal surgery, urologic surgery, orthopedic surgery, neurosurgery, gynecologic surgery, or other surgical disciplines).
- Intervention/Exposure: Occurrence of one or more postoperative complications as defined by individual studies. No restrictions were placed on complication types or definitions to capture the full heterogeneity of FTR operationalization.
- Comparator: Studies with or without comparator groups were eligible. Comparators, when present, included different hospital types (teaching vs. non-teaching, high-volume vs. low-volume), different care processes (rapid response teams vs. standard care, different time-to-intervention thresholds), or different patient populations (stratified by age, comorbidity, or complication severity).
- Outcome: Failure to rescue, defined as death following a postoperative complication, reported as either the primary or secondary outcome. FTR could be operationalized as a structural indicator (hospital/system-level characteristics associated with FTR rates), process indicator (care pathways or interventions affecting FTR), or clinical outcome (patient-level mortality stratified by complication type or patient factors).
- Study design: Clinical studies including prospective or retrospective cohort studies, case-control studies, comparative observational studies, clinical trials (randomized or non-randomized), and multicenter registry-based studies. Case reports, editorials, commentaries, letters, conference abstracts, and narrative reviews were excluded.

### Data sources and search strategy

A comprehensive literature search was performed in May 2024 using PubMed and PubMed Central databases. Two complementary search queries were designed to maximize retrieval of clinical surgical studies reporting FTR: **Query 1**: ((“failure” [All Fields] OR “failures” [All Fields]) AND (“rescue” [All Fields] OR “rescued” [All Fields] OR “rescues” [All Fields] OR “rescuing” [All Fields])) AND ((y_5[Filter]) AND (classical article[Filter] OR clinical study[Filter] OR clinical trial[Filter] OR multicenter study[Filter] OR observational study[Filter] OR randomized controlled trial[Filter]) AND (humans[Filter]) AND (english[Filter])); **Query 2**: (“ftr” [All Fields]) AND ((y_5[Filter]) AND (clinical study[Filter] OR clinical trial[Filter] OR multicenter study[Filter] OR observational study[Filter] OR randomized controlled trial[Filter]) AND (humans[Filter]) AND (English[Filter])).

Query 1 was designed to maximize sensitivity by capturing all variations of the phrase “failure to rescue” using Boolean operators: (“failure” [All Fields] OR “failures” [All Fields]) AND (“rescue” [All Fields] OR “rescued” [All Fields] OR “rescues” [All Fields] OR “rescuing” [All Fields]). This broad approach ensured capture of the concept regardless of exact phrasing (e.g., “failure-to-rescue,” “failure to rescue,” “rescue of patients who failed initial treatment”).

Query 2 specifically targeted the acronym “FTR,” which is increasingly used in surgical quality literature. While this query is narrower, it captured studies where FTR was used as a technical term without necessarily repeating the full phrase throughout the manuscript.

Search filters limited results to human studies, English language, and clinical study types published between January 1, 2019, and May 31, 2024.

Inclusion criteria were: clinical studies (observational, comparative, randomized trials) reporting FTR as an outcome; surgical populations (any subspecialty); publication date: 2019–2024; English language; and reporting of patient-level or system-level FTR data. Exclusion criteria were: case reports, editorials, letters, conference abstracts; studies not focused on FTR or not presenting clinical data; non-surgical populations; and duplicate publications.

Duplicate references were removed semi-automatically using Microsoft Excel. Two independent reviewers (CV, LL) screened titles and abstracts against eligibility criteria. Full-text articles were retrieved for potentially eligible studies, and final inclusion was determined by consensus. Disagreements were resolved through discussion with a third reviewer (AB).

For each included manuscript, the following data were extracted using a standardized form: Bibliographic information [Author(s), year, journal name, Journal Impact Factor (JIF) from the most recent Journal Citation Reports (15)]; Study characteristics (study design, country of corresponding author, surgical subspecialty, sample size); FTR operationalization [Outcome hierarchy: primary or secondary outcome; Indicator type: Structure -hospital/system characteristics, Process -care pathways, protocols, interventions-, or Clinical Outcome -patient-level mortality-, according to Donabedian's framework (3)]; Bibliometric data [citations from Scopus (16) and Google Scholar (17) -retrieved via DOI search; Altmetric Attention Score (18); Keywords (Medical Subject Headings (MeSH) terms assigned in PubMed].

### Risk of bias assessment

All included studies were assessed for methodological quality using the Newcastle-Ottawa Scale (NOS) (19), which evaluates cohort and case-control studies across three domains: selection of study groups, comparability of groups, and ascertainment of outcome or exposure. Studies were rated as low, moderate, or high risk of bias.

### Outcomes of interest

Primary outcomes of interest were: temporal trends in FTR clinical studies (2019–2024); FTR as primary vs. secondary outcome; and FTR operationalization: structure, process, or clinical outcome indicator. Secondary outcomes included: geographic distribution of contributing authors (by country); surgical subspecialty representation; Bibliometric impact: Scopus citations, Google Scholar citations, Altmetric scores and MeSH term frequency analysis.

### Data synthesis and statistical analysis

Descriptive statistics (mean, standard deviation, median, range, frequencies, percentages) were calculated for all variables. Bibliometric indices were compared across indicator categories (structure/process/outcome) and surgical subspecialties using one-way analysis of variance (ANOVA). Statistical significance was set at *p* < 0.05. All analyses were performed using Microsoft Excel and R (version 4.x).

## Results

### Study selection

**Figure 1:**
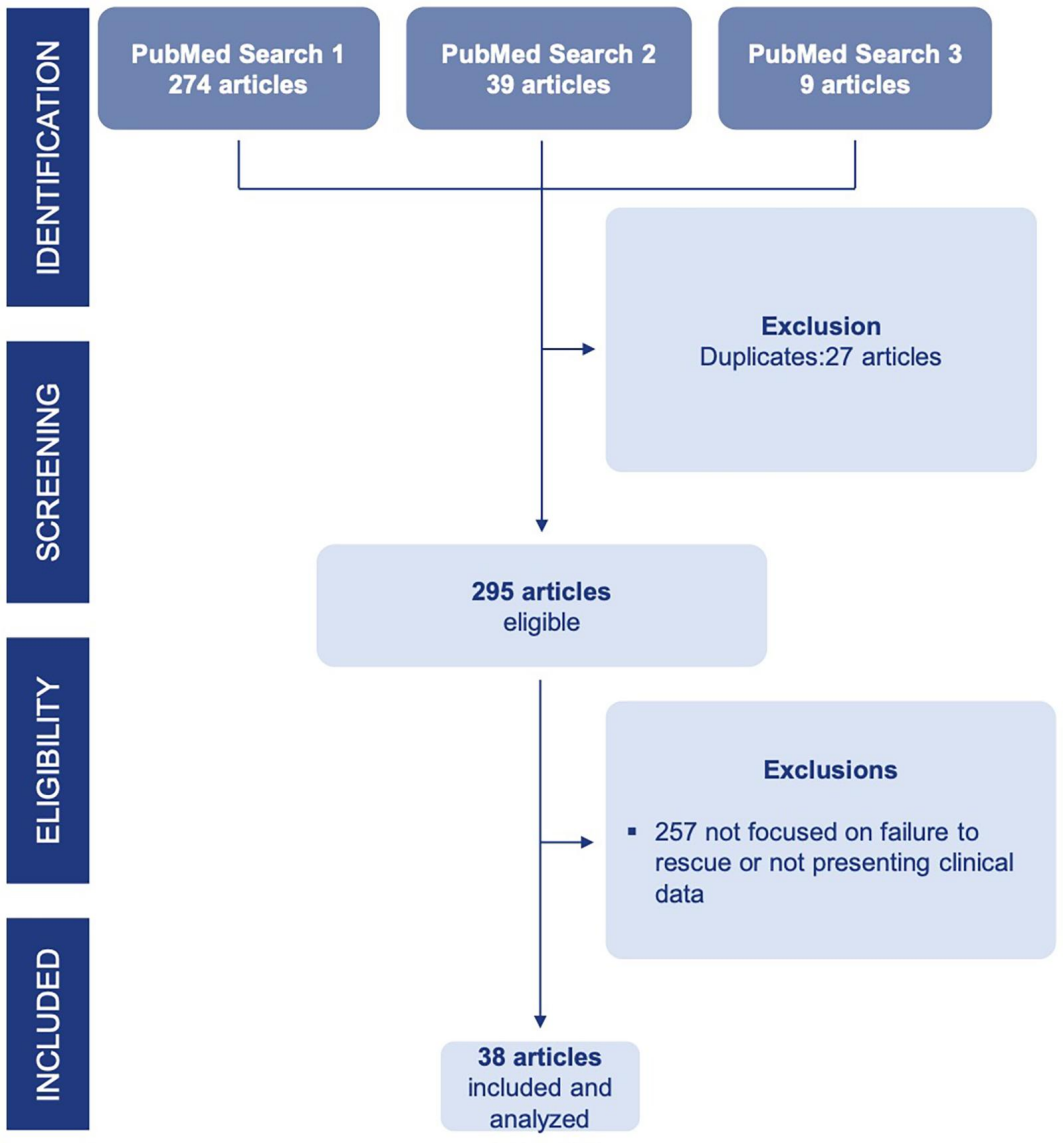
PRISMA flow-chart.

The initial search yielded 322 articles. After removing 27 duplicates, 295 articles underwent screening. Of these, 257 were excluded because they did not focus on FTR or provide clinical data. The remaining 38 articles met all inclusion criteria and were included in the systematic review (Figure 1). The full list of studies included in the analysis is provided in Supplementary Materials. All studies were retrospective and 3 manuscripts were based on multinational datasets.

### Risk of bias assessment

**Figure 2:**
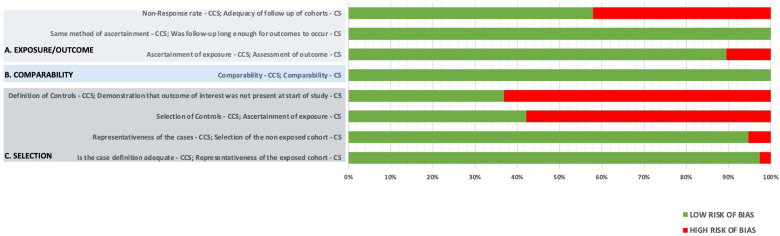
Newcastle-Ottawa scale of included studies. **(A)** Exposure/outcome. **(B)** Comparability. **(C)** Selection.

Overall, the included studies demonstrated a moderate-to-low risk of bias. Most studies (>90%) adequately defined exposure and outcome, ensured sufficient follow-up duration, and used comparable cohorts. However, approximately 30% of studies exhibited a high risk of bias in the domains of control group definition and exposure ascertainment, reflecting the retrospective nature of most FTR research (Figure 2).

### Temporal trends and study characteristics

Publication output was modest and showed limited growth. Studies were distributed as follows: 2019 (*n* = 9, 23.7%), 2020 (*n* = 9, 23.7%), 2021 (*n* = 11, 28.9%), 2022 (*n* = 5, 13.2%), and 2023–2024 (*n* = 4, 10.5%). The decline after 2021 is notable given the increasing emphasis on quality metrics during the COVID-19 pandemic period.

The majority of studies were large, multicenter retrospective cohorts. The median sample size was 29,482 patients (range: 57–20,922,025), reflecting the use of national registries and administrative databases. Mean sample size was 641,054 patients (SD: 3,382,825), driven by several very large registry-based studies (Table 1).

**Table 1:** Failure to rescue in clinical literature.

Articles over time	*n*	%
2019	9	23.7
2020	9	23.7
2021	11	28.9
2022	5	13.2
2023–2024	4	10.5
Number of patients		
Mean (SD)	641,054.2	3,382,824.9
Median (range)	29,481.5	(57–20,922,025)

Failure to rescue as outcome of interest	*n*	%
First	27	71.0
Second	11	29.0

Failure to rescue as an indicator	*n*	%
Clinical Outcome	10	26.3
Process	11	28.9
Structure	17	44.8

Failure to rescue definition (numerator)	*n*	%
Death after postoperative complication	34	89.5
Mortality for any cause	2	5.3
Not defined	2	5.3

Failure to rescue definition (denominator)—severity of complications	*n*	%
Any	15	39.5
Moderate (i.e., >grade 2)	1	2.6
Major	21	55.3
Not defined	1	2.6

Failure to rescue definition (denominator)—time-frame of complications	*n*	%
2-days	1	2.6
30-days	14	36.8
90-days	7	18.4
In-hospital	7	18.4
Not defined	9	23.7

The extreme skewness in sample sizes due to the mega-registry studies provides statistical power for detecting small effect sizes and enable national-level benchmarking. These datasets also introduce considerations on the administrative data limitations, and the heterogeneity of national scale studies.

Table 1 reports also the heterogeneity in FTR definitions across included studies (*n* = 38). This heterogeneity has important implications. Studies using in-hospital mortality may underestimate FTR compared to those using 30-day mortality, as some deaths occur post-discharge. The number and type of complications included in the denominator directly affect FTR rates—broader complication definitions yield lower FTR percentages, while restricting to major complications increases observed FTR. Additionally, 23.7% of studies did not clearly specify the time-frame of complications, limiting interpretability.

### FTR operationalization

Across the 38 included studies, failure to rescue was most often placed at the center of the analytic framework, serving as the primary outcome in 27 manuscripts (71.0%) and as a secondary endpoint in the remaining 11 (29.0%). Rather than being treated as a marginal or exploratory variable, FTR is increasingly the key lens through which postoperative safety and system performance are evaluated. When mapped onto Donabedian's framework, almost half of the studies (44.8%, *n* = 17) used FTR as a structural indicator, linking it to hospital-level characteristics such as case volume, teaching status, nurse-to-patient ratios, intensive care capacity, or geographic setting. A smaller but important group of investigations (28.9%, *n* = 11) conceptualized FTR as a process measure, embedding it within specific interventions and care pathways, including rapid response systems, enhanced recovery protocols, or time-sensitive reinterventions. Finally, in just over a quarter of the manuscripts (26.3%, *n* = 10), FTR was framed primarily as a clinical outcome, reported as a patient-level mortality metric, and often stratified according to individual risk profiles or disease categories. This distribution suggests that FTR is most commonly used to characterize *where* care is delivered, with less emphasis on *how* care is delivered or *what* patient-level factors influence rescue success.

### Geographic distribution

**Figure 3:**
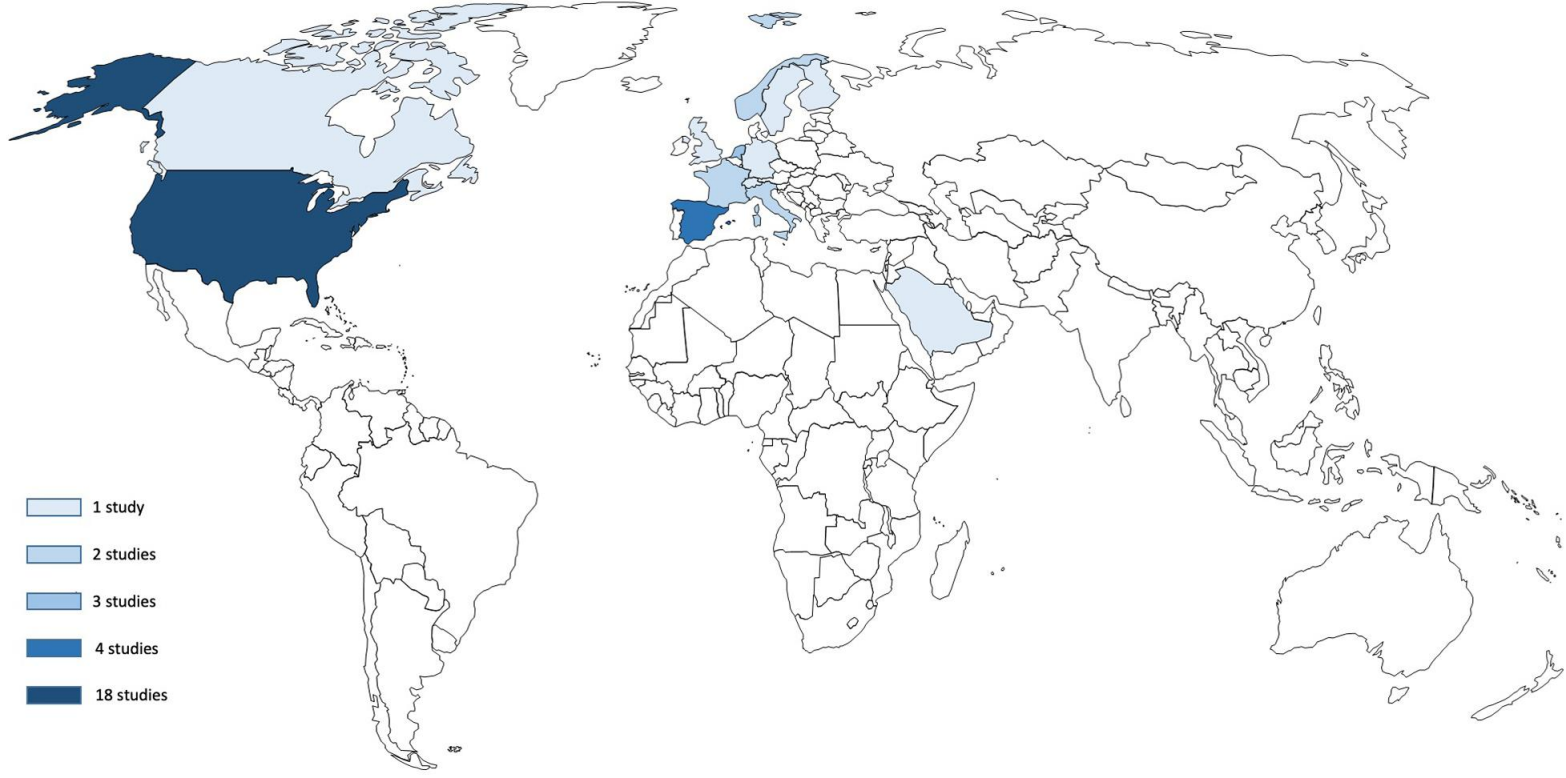
World map distribution of clinical studies on failure to rescue.

The geographic distribution of FTR research was highly concentrated in high-income countries (Figure 3). The United States accounted for nearly half of all studies (*n* = 18, 47.4%), followed by Spain (*n* = 4, 10.5%), the Netherlands (*n* = 3, 7.9%), and France, Italy, and Norway (*n* = 2 each, 5.3%). Single studies originated from Canada, England, Finland, Germany, Saudi Arabia, Sweden, and Switzerland (2.6% each). No studies from low- or middle-income countries in Africa, Asia (except Saudi Arabia), or Latin America met the inclusion criteria.

### Surgical subspecialty representation

The published literature was heavily skewed toward abdominal surgery and closely related subspecialties. Emergency surgery and trauma accounted for the largest share of studies (18.4%, *n* = 7), followed by cardiovascular surgery (15.8%, *n* = 6), and hepatobiliary-pancreatic, colorectal, and thoracic surgery, each contributing five manuscripts (13.2% each). General abdominal surgery (7.9%, *n* = 3) and upper gastrointestinal surgery (5.3%, *n* = 2) were less frequently represented, while gynecologic, cytoreductive, vascular, and miscellaneous procedures together comprised a further 13.2% of the sample. Taken together, emergency surgery and the various abdominal subspecialties accounted for 58% of all publications, underscoring that FTR research has largely focused on clinical settings with high complication burdens and complex rescue dynamics.

### Bibliometric impact

**Figure 4:**
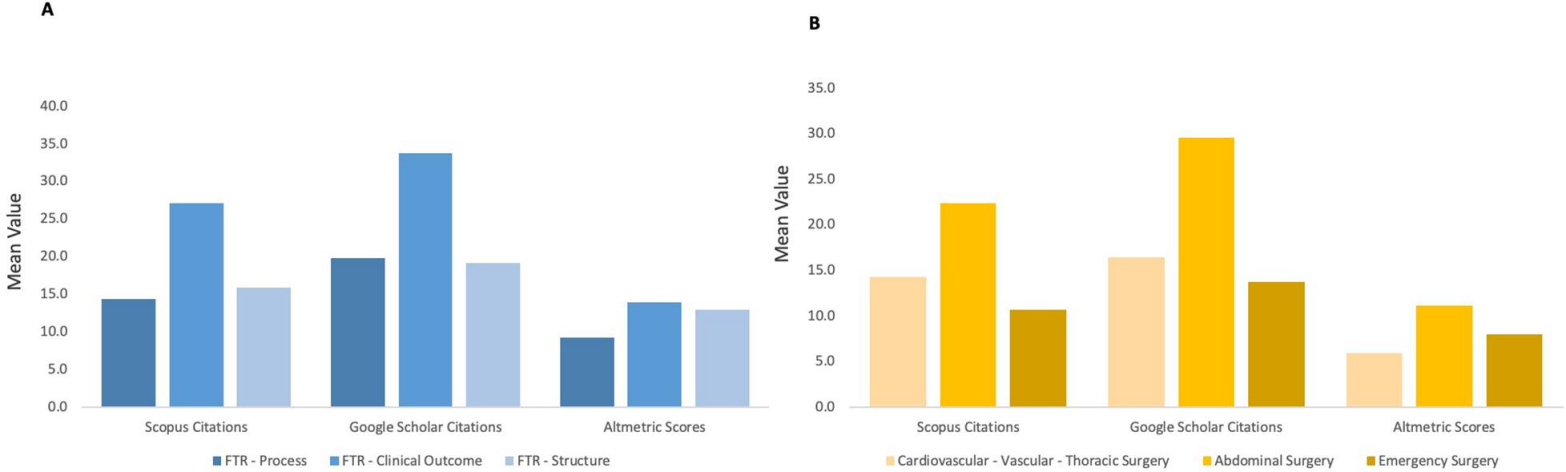
Bibliometric indexes of clinical studies on failure to rescue (FTR). **(A)** FTR as an indicator. **(B)** FTR in different sub-specialties.

Despite the recent publication period, the included studies achieved substantial scholarly impact (Table 2 and Figure 4). Mean citations were 18.4 (SD: 34.1) in Scopus and 23.2 (SD: 43.4) in Google Scholar, with medians of 8.0 and 9.5, respectively. The mean Altmetric Attention Score was 12.1 (SD: 21.4), indicating moderate online engagement. The majority of studies (76%) were published in high-impact, first-quartile surgical journals (mean JIF: 6.2, SD: 4.7; median: 4.1; range: 1.6–16.9).

**Table 2:** Bibliometric indexes.

Bibliometric Index	Mean/Median value	Standard deviation/range
Mean (SD)	6.2	4.7
Median (range)	4.1	1.6–16.9
Scopus Citations		
Mean (SD)	18.4	34.1
Median (range)	8.0	(0–195)
Google Scholar Citations		
Mean (SD)	23.2	43.4
Median (range)	9.5	(0–242)
Altmetric Score		
Mean (SD)	12.1	21.4
Median (range)	4.0	(0–111)

One-way ANOVA revealed no significant differences in bibliometric impact across indicator categories (Scopus: *F* = 0.44, *p* = 0.65; Google Scholar: *F* = 0.39, *p* = 0.68; Altmetric: *F* = 0.14, *p* = 0.87) or surgical subspecialties (Scopus: *F* = 0.32, *p* = 0.72; Google Scholar: *F* = 0.43, *p* = 0.65; Altmetric: *F* = 0.49, *p* = 0.62). This suggests that FTR studies, regardless of how they operationalize the metric or which subspecialty they address, achieve comparable scholarly impact—a finding consistent with a field that is still small and relatively homogeneous in terms of methodological approach and target audience.

### MeSH term analysis

The most frequently assigned MeSH terms reflected the nature of included studies (Table 3): “Retrospective Studies” appeared in 78.9% of articles, confirming the predominance of observational designs. “Postoperative Complications” (in various subheadings) was central, as expected, and “Hospital Mortality” reflected the focus on system-level outcomes. Notably, “Failure to Rescue, Health Care” as a standalone MeSH term appeared in only 15 studies (39.5%), suggesting inconsistent indexing and possible challenges in literature retrieval.

**Table 3:** Top-10 MeSH terms.

**MeSH terms**	**N**	**MeSH terms (grouped)**	**N3**
Humans	38	Postoperative Complications	47
Retrospective Studies	30	Humans	38
Female	23	Retrospective Studies	30
Male	22	Female	23
Postoperative Complications/epidemiology	19	Male	22
Aged	16	Hospital Mortality	20
Middle Aged	14	Aged	16
Hospital Mortality	12	Failure to Rescue, Health Care	15
Postoperative Complications/etiology	12	Middle Aged	14
Adult	11	United States	12

## Discussion

This systematic review provides the first comprehensive appraisal of how failure to rescue has been operationalized and disseminated in recent surgical clinical studies. Three key findings emerge. First, despite FTR's conceptual prominence as a quality metric, the volume of high-quality clinical surgical research remains limited: only 38 studies met inclusion criteria over a five-year period, with publication rates declining after 2021. Second, FTR is predominantly used as a structural indicator (44.8%), examining hospital and system characteristics, rather than as a process indicator that evaluates care pathways or as a clinical outcome measure stratified by patient factors. Third, the FTR evidence base is geographically and subspecialty-constrained: nearly 90% of studies originate from the USA and Europe, and over half address abdominal or emergency surgery, leaving major gaps in other surgical domains and in low- and middle-income countries.

Donabedian's framework posits that healthcare quality can be assessed through structure (resources and organizational features), process (what is done to and for patients), and outcome (the effects of care on health status) (3). Our findings show that FTR research is disproportionately structural: studies correlate FTR with hospital volume, teaching status, nurse staffing, and geographic location. This reflects the feasibility of leveraging administrative data and national registries to conduct large-scale, retrospective analyses (20, 21).

However, structural associations provide limited actionable guidance for quality improvement. Understanding *that* high-volume centers have lower FTR does not explain *why*—what specific processes (e.g., earlier recognition, faster escalation, protocol adherence) drive rescue success. Process studies (28.9% of our sample) begin to address this gap by examining interventions such as rapid response teams, time to reoperation, and enhanced recovery protocols (22, 23). Yet process studies remain in the minority and are often single-center or limited by a lack of experimental control.

Clinical outcome studies (26.3%) focus on patient-level factors (age, comorbidity, complication type) that modify FTR risk. While important for risk stratification, these studies do not directly inform system-level interventions. The relative scarcity of process-oriented and interventional FTR studies represents a critical gap: FTR must transition from a surveillance metric to a target for active quality improvement (24, 25).

The near-total dominance of high-income countries (the USA at 47.4%, Europe at 42.1%) in FTR research is striking. This concentration likely reflects data infrastructure (national surgical registries, linkage to mortality records) and research funding, but it raises serious questions about the generalizability of FTR as a global quality metric. Low- and middle-income countries (LMICs), where surgical volume and complication rates are rising, and systems for recognizing and escalating care may be less robust, are entirely absent from the literature (26, 27). Surgical safety initiatives, such as the WHO Safe Surgery Checklist, have demonstrated impact in resource-limited settings (28); FTR could serve as a complementary metric, but only if validated and adapted for LMIC contexts.

Similarly, the concentration of FTR studies in abdominal and emergency surgery (58% combined) reflects the high complication burden in these fields but leaves other major surgical subspecialties underrepresented. Orthopedic surgery, neurosurgery, and urologic surgery—each with substantial volumes and postoperative risks—are nearly absent from the FTR literature. Expanding FTR research across the full spectrum of surgical subspecialties is essential for understanding whether rescue dynamics differ by operative context and patient population (29, 30).

Cardiovascular surgery accounted for 15.8% (*n* = 6) of included studies, representing the second most-studied surgical subspecialty after emergency surgery. This concentration reflects both the high complication burden in cardiac surgery and the field's longstanding engagement with quality measurement and risk-adjusted outcome reporting. Future cardiovascular FTR research should expand beyond volume-outcome analyses to test specific interventions, examine FTR across the full spectrum of cardiac procedures, and investigate the role of team composition and care protocols in rescue success.

The high bibliometric impact of included studies—mean citations of 18.4 (Scopus) and 23.2 (Google Scholar) within 2–5 years, publication in first-quartile journals (mean JIF 6.2)—suggests that FTR research resonates with the surgical and quality-improvement communities. However, the lack of significant differences in impact across indicator types or subspecialties indicates that the field is still too small and methodologically homogeneous to show differentiation. As FTR research expands and diversifies, future bibliometric analyses may reveal whether process-oriented or interventional studies achieve greater translational impact.

The moderate Altmetric scores (mean: 12.1) suggest that FTR studies receive some attention beyond traditional academic channels (social media, policy documents, news outlets), but uptake is not yet widespread. Strategies to enhance dissemination—such as plain-language summaries, integration into quality dashboards, and engagement with patient advocacy groups—may accelerate the translation of FTR evidence into practice (31).

Our findings complement and extend prior reviews. Wells *et al*. (2024) documented heterogeneity in FTR definitions across 295 studies, identified 131 distinct complications used in FTR calculations, and called for standardization (4). Our review, by focusing on *clinical* surgical studies from 2019 to 2024, shows that definitional heterogeneity persists and that the volume of new evidence is modest. Sheetz et al. provided a conceptual framework for FTR as a surgical quality indicator, emphasizing the need for systems-level interventions (13). Our systematic appraisal empirically demonstrates that such systems-level (process) studies remain rare.

Recent empirical work has begun to address some gaps. Schwappach et al. demonstrated substantial between-hospital variation in FTR after major surgery, even after risk adjustment, highlighting the potential for quality improvement (32). Studies in cardiac and emergency surgery have identified modifiable factors, such as time to intervention and multidisciplinary team structure (9, 11, 33). Our review situates these advances within the broader landscape, showing that they represent important but isolated contributions to a field that requires greater coordination and standardization.

On this basis, a consensus core set of complications for FTR calculation, stratified by surgical subspecialty, is urgently needed to enable valid comparisons across studies and institutions (4, 5). International surgical societies and quality organizations should convene expert panels to develop and disseminate standardized definitions of FTR.

Also, FTR research must extend to low- and middle-income countries, where surgical volume is growing rapidly and where systems for recognizing and escalating complications may face different resource constraints. International collaboratives, such as GlobalSurg or the African Organization for Research and Training in Cancer, offer potential platforms (26, 34).

The complete absence of studies from low- and middle-income countries (LMICs) in our review is particularly concerning. Several structural factors explain why FTR is difficult to measure and study in resource-limited settings: limited electronic health records, limited post-discharge mortality tracking and inconsistent complication documentation, and resource constraints for follow-up.

Despite these challenges, FTR could be a valuable metric in LMICs precisely because it highlights systems-level deficiencies in recognizing and responding to complications. International collaboratives such as GlobalSurg have demonstrated the feasibility of prospective surgical outcomes research in resource-limited settings using simplified data collection tools and mobile technology (26). Adapting FTR for LMIC contexts may require modified definitions (e.g., in-hospital FTR only, focus on preventable deaths), use of verbal autopsy for mortality ascertainment, and integration with existing surgical registries. Pilot studies establishing feasibility and validity of FTR measurement in LMICs are urgently needed.

Future research should also shift from descriptive, structural analyses to experimental or quasi-experimental studies that test interventions to reduce FTR—such as enhanced surveillance protocols, early warning systems, structured escalation pathways, and multidisciplinary rapid response teams (22–23, 25). Embedding FTR endpoints in quality-improvement collaboratives and pragmatic trials will be essential (35). Finally, surgical subspecialties beyond abdominal and emergency surgery should adopt FTR as a routine outcome metric in registries, trials, and quality dashboards. This will enable comparative effectiveness research and subspecialty-specific benchmarking (29, 30).

Strengths of this review include adherence to PRISMA guidelines, systematic risk-of-bias assessment, comprehensive bibliometric analysis, and focus on the most recent literature (2019–2024). By restricting inclusion to clinical surgical studies, we provide a targeted appraisal of how FTR is used in practice-relevant research.

Limitations include restriction to PubMed and PubMed Central databases and English-language publications, which may have excluded relevant studies indexed elsewhere or published in other languages. We did not register a protocol prospectively, though methods were specified *a priori* and reported transparently. The relatively small number of included studies (*n* = 38) limited subgroup analyses and precluded meta-analysis. Also, the study protocol was registered retrospectively rather than prospectively, which may limit protection against selective outcome reporting bias. However, all methods were specified *a priori* and applied systematically throughout the review process. It has to be acknowledged that the search was restricted to PubMed and PubMed Central (PMC). While PRISMA guidelines recommend searching multiple databases, this decision was based on several considerations. Although PubMed provides comprehensive coverage of surgical and healthcare quality literature, we acknowledge that this approach may have missed relevant studies published in subspecialty journals not indexed in PMC, particularly European and Asian surgical journals, and may have reduced sensitivity for recently published articles with indexing delays. Future systematic reviews should employ multi-database search strategies, include non-English databases, and incorporate reference list screening (snowballing) to ensure comprehensive capture. The limitation is particularly important given our finding of geographic concentration—expanding database coverage might reveal more diverse international contributions. No formal grey literature search (e.g., conference proceedings, institutional repositories, trial registries) was performed, as our focus was on peer-reviewed clinical studies with full methodological reporting. Similarly, we did not perform systematic reference list screening (snowballing) or forward citation searching, as these methods were deemed beyond the scope of this review given the specificity of FTR as a keyword.

Finally, bibliometric indices are influenced by time since publication; studies from 2023 to 2024 had less time to accrue citations, though the ANOVA analysis found no significant effect of publication year on impact.

## Conclusions

Failure to rescue is a critical quality and safety metric with the potential to drive meaningful improvements in surgical care. However, current FTR research is limited in volume, geographically concentrated, subspecialty-skewed, and predominantly structural rather than process-oriented. To advance from surveillance to action, the field requires standardized definitions, expansion to underrepresented regions and subspecialties, and a shift toward interventional studies that test strategies to improve rescue. The high bibliometric impact of existing studies demonstrates the field's relevance; what is needed now is scale, diversity, and a commitment to translating evidence into practice.

## Data Availability

The raw data supporting the conclusions of this article will be made available by the authors, without undue reservation.
