# Supplementary material for: Failure-to-rescue in surgical practice: a systematic review and critical appraisal of recent clinical studies
Source: Front Surg. 2026 May 19;13:1827601. doi: 10.3389/fsurg.2026.1827601 (PMC13226588; doi:10.3389/fsurg.2026.1827601)
Supplement: Supplementary file 1 [file Supplementaryfile1.docx]

**Supplementary Materials - Index**

| **Type - Supplementary Tables** |  |
| --- | --- |
| Supplementary Table 1 | *page 2* |
| Supplementary Table 2 | *page 3* |
| Supplementary Table 3 | *page 4* |
| **Type - Supplementary Figure** |  |
| Supplementary Figure 1 | *page 9* |
| **Supplementary References -** |  |
| Manuscripts included in the analysis | *page 10* |

**Supplementary Table 1. Geography and sub-specialties of manuscripts included in the systematic review**

| Country | n | % |
| --- | --- | --- |
| Canada | 1 | 2.6 |
| England | 1 | 2.6 |
| Finland | 1 | 2.6 |
| France | 2 | 5.3 |
| Germany | 1 | 2.6 |
| Italy | 2 | 5.3 |
| The Netherlands | 3 | 7.9 |
| Norway | 2 | 5.3 |
| Saudi Arabia | 1 | 2.6 |
| Spain | 4 | 10.5 |
| Sweden | 1 | 2.6 |
| Switzerland | 1 | 2.6 |
| USA | 18 | 47.4 |
| Subspecialty | **n** | **%** |
| Abdominal Surgery | 3 | 7.9 |
| Cardiovascular Surgery | 6 | 15.8 |
| Colorectal Surgery | 5 | 13.2 |
| Cytoreductive Surgery | 1 | 2.6 |
| Emergency Surgery & Trauma | 7 | 18.4 |
| Gynecologic Surgery | 1 | 2.6 |
| Hepato-bilio-pancreatic Surgery | 5 | 13.2 |
| Miscellaneous | 2 | 5.3 |
| Thoracic Surgery (including transplants) | 5 | 13.2 |
| Upper Gastrointestinal Surgery | 2 | 5.3 |
| Vascular Surgery | 1 | 2.6 |

**Supplementary Table 2. ANOVA analysis of bibliometric indexes**

| **Clinical outcome *vs* process *vs* structure** | | | | | | | | | | |  |  |
| --- | --- | --- | --- | --- | --- | --- | --- | --- | --- | --- | --- | --- |
| **Scopus** | **DF** | | | **Sum of Square** | | **Mean Square** | | **F Statistic** | | **P-value** |  |  |
| **Groups** (between groups) | 2 | | | 1048.9261 | | 524.463 | | 0.43662 | | 0. 649691 |  |  |
| **Error** (within groups) | 35 | | | 42041.916 | | 1201.1976 | |  | |  |  |  |
| **Total** | 37 | | | 43090.8421 | |  | |  | |  |  |  |
|  |  |  |  |  |  |  |  |  |  |  |  |  |
| **Google Scholar** | **DF** | | | **Sum of Square** | | **Mean Square** | | **F Statistic** | **P-value** | |  |  |
| **Groups** (between groups) | 2 | | | 1532.7095 | | 766.3547 | | 0.39444 | 0. 677015 | |  |  |
| **Error** (within groups) | 35 | | | 68001.0011 | | 1942.8857 | |  |  | |  |  |
| **Total** | 37 | | | 69533.7105 | |  | |  |  | |  |  |
|  |  |  |  |  |  |  |  |  |  |  |  |  |
| **Altmetric** | **DF** | | | **Sum of Square** | **Mean Square** | | | **F Statistic** | **P-value** | |  |  |
| **Groups** (between groups) | 2 | | | 136.4621 | 68.231 | | | 0.14194 | 0. 868168 | |  |  |
| **Error** (within groups) | 35 | | | 16824.3011 | 480.6943 | | |  |  | |  |  |
| **Total** | 37 | | | 16960.7632 | 458.399 | | |  |  | |  |  |
| **Cardiovascular *vs* abdominal *vs* emergency** | | | | | | | | | | | | |
| **Scopus** | | **DF** | **Sum of Square** | | **Mean Square** | | | **F Statistic** | **P-value** | | | |
| **Groups** (between groups) | | 2 | 827.2571 | | 413.6286 | | | 0.3249 | 0.7249 | | | |
| **Error** (within groups) | | 32 | 40737.4291 | | 1273.0447 | | |  |  | | | |
| **Total** | | 34 | 41564.6862 | | 1222.4908 | | |  |  | | | |
| **Google Scholar** | | **DF** | **Sum of Square** | | **Mean Square** | | **F Statistic** | | **P-value** | | |  |
| **Groups** (between groups) | | 2 | 1769.26 | | 884.63 | | 0.4345 | | 0.6513 | | |  |
| **Error** (within groups) | | 32 | 65148.2832 | | 2035.8839 | |  | |  | | |  |
| **Total** | | 34 | 66917.5433 | | 1968.163 | |  | |  | | |  |
| **Altmetric** | | **DF** | **Sum of Square** | | **Mean Square** | | | **F Statistic** | **P-value** | | | |
| **Groups** (between groups) | | 2 | 190.4762 | | 95.2381 | | | 0.4863 | 0.6194 | | | |
| **Error** (within groups) | | 32 | 6266.6668 | | 195.8333 | | |  |  | | | |
| **Total** | | 34 | 6457.143 | | 189.916 | | |  |  | | | |

**Supplementary Table 3. MeSH terms**

| **MeSH** | **n** | % |
| --- | --- | --- |
| Humans | 38 |  |
| Retrospective Studies | 30 |  |
| Female | 23 |  |
| Male | 22 |  |
| Postoperative Complications / epidemiology | 19 |  |
| Aged | 16 |  |
| Middle Aged | 14 |  |
| Hospital Mortality | 12 |  |
| Postoperative Complications / etiology | 12 |  |
| Adult | 11 |  |
| Failure to Rescue, Health Care / statistics & numerical data | 9 |  |
| Risk Factors | 9 |  |
| Hospital Mortality / trends | 8 |  |
| United States / epidemiology | 8 |  |
| Postoperative Complications / mortality | 7 |  |
| Hospitals | 6 |  |
| Incidence | 6 |  |
| Adolescent | 5 |  |
| Failure to Rescue, Health Care | 5 |  |
| Follow-Up Studies | 5 |  |
| Survival Rate / trends | 5 |  |
| Aged, 80 and over | 4 |  |
| Cohort Studies | 4 |  |
| Hospitals, High-Volume / statistics & numerical data | 4 |  |
| Prospective Studies | 4 |  |
| Quality Improvement | 4 |  |
| Registries | 4 |  |
| Time Factors | 4 |  |
| Treatment Outcome | 4 |  |
| United States | 4 |  |
| Young Adult | 4 |  |
| Child | 3 |  |
| Child, Preschool | 3 |  |
| Cross-Sectional Studies | 3 |  |
| Elective Surgical Procedures / adverse effects | 3 |  |
| Hospitals / statistics & numerical data | 3 |  |
| Hospitals, Low-Volume / statistics & numerical data | 3 |  |
| Infant | 3 |  |
| Infant, Newborn | 3 |  |
| Intensive Care Units | 3 |  |
| Postoperative Complications / therapy | 3 |  |
| Aortic Aneurysm, Abdominal / mortality | 2 |  |
| Aortic Aneurysm, Abdominal / surgery | 2 |  |
| Carcinoma, Hepatocellular / mortality | 2 |  |
| Carcinoma, Hepatocellular / surgery | 2 |  |
| Cardiac Catheterization / adverse effects | 2 |  |
| Cardiac Surgical Procedures / adverse effects | 2 |  |
| Colonic Neoplasms / surgery | 2 |  |
| COVID-19 / epidemiology | 2 |  |
| Databases, Factual | 2 |  |
| Digestive System Surgical Procedures / adverse effects | 2 |  |
| Emergencies | 2 |  |
| Esophagectomy / adverse effects | 2 |  |
| Europe / epidemiology | 2 |  |
| Heart Defects, Congenital / mortality | 2 |  |
| Heart Defects, Congenital / surgery | 2 |  |
| Hospitals, High-Volume | 2 |  |
| Liver Neoplasms / mortality | 2 |  |
| Liver Neoplasms / surgery | 2 |  |
| Lung Transplantation | 2 |  |
| Pandemics | 2 |  |
| Patient Readmission / statistics & numerical data | 2 |  |
| Postoperative Complications | 2 |  |
| Postoperative Complications / diagnosis | 2 |  |
| Postoperative Complications / surgery | 2 |  |
| Prognosis | 2 |  |
| Propensity Score | 2 |  |
| Quality Indicators, Health Care | 2 |  |
| Risk Assessment / methods | 2 |  |
| Alberta | 1 |  |
| Anastomotic Leak / epidemiology | 1 |  |
| Anastomotic Leak / etiology | 1 |  |
| Anemia / blood | 1 |  |
| Anemia / complications | 1 |  |
| Anemia / diagnosis | 1 |  |
| Anemia / drug therapy | 1 |  |
| Aorta, Thoracic / surgery | 1 |  |
| Aortic Aneurysm, Thoracic / surgery | 1 |  |
| Aortic Dissection | 1 |  |
| Asia / epidemiology | 1 |  |
| Australia / epidemiology | 1 |  |
| Benchmarking | 1 |  |
| Bile Duct Neoplasms / mortality | 1 |  |
| Bile Duct Neoplasms / surgery | 1 |  |
| Blood Transfusion / statistics & numerical data | 1 |  |
| Blood Vessel Prosthesis Implantation / adverse effects | 1 |  |
| Blood Vessel Prosthesis Implantation / methods | 1 |  |
| Cardiac Catheterization / mortality | 1 |  |
| Cardiac Catheterization / statistics & numerical data | 1 |  |
| Cardiac Catheterization / trends | 1 |  |
| Cardiac Surgical Procedures / methods | 1 |  |
| Cardiac Surgical Procedures / mortality | 1 |  |
| Cardiac Surgical Procedures / standards | 1 |  |
| Cardiac Surgical Procedures / trends | 1 |  |
| Cardiovascular Diseases / complications | 1 |  |
| Cardiovascular Diseases / surgery | 1 |  |
| Cesarean Section / mortality | 1 |  |
| Cholangiocarcinoma / mortality | 1 |  |
| Cholangiocarcinoma / surgery | 1 |  |
| Clinical Audit | 1 |  |
| Colectomy | 1 |  |
| Colorectal Neoplasms / therapy | 1 |  |
| Colorectal Surgery / adverse effects | 1 |  |
| Coma / surgery | 1 |  |
| Comorbidity | 1 |  |
| Coronary Artery Bypass / adverse effects | 1 |  |
| Digestive System Surgical Procedures / mortality | 1 |  |
| Elective Surgical Procedures | 1 |  |
| Elective Surgical Procedures / mortality | 1 |  |
| Emergency Service, Hospital / statistics & numerical data | 1 |  |
| Emergency Treatment / mortality | 1 |  |
| Endovascular Procedures / adverse effects | 1 |  |
| Esophageal Neoplasms / mortality | 1 |  |
| Esophageal Neoplasms / surgery | 1 |  |
| Failure to Rescue, Health Care / trends | 1 |  |
| Frail Elderly / statistics & numerical data | 1 |  |
| Frailty / complications | 1 |  |
| Frailty / diagnosis | 1 |  |
| Frailty / epidemiology | 1 |  |
| Gastrectomy / adverse effects | 1 |  |
| Gastrectomy / methods | 1 |  |
| General Surgery | 1 |  |
| General Surgery / statistics & numerical data | 1 |  |
| Geriatric Assessment / methods | 1 |  |
| Germany / epidemiology | 1 |  |
| Health Expenditures | 1 |  |
| Health Services Accessibility / economics | 1 |  |
| Health Workforce / statistics & numerical data | 1 |  |
| Healthcare Disparities / trends | 1 |  |
| Heart Defects, Congenital / diagnosis | 1 |  |
| Heart Diseases / mortality | 1 |  |
| Heart Valve Diseases / mortality | 1 |  |
| Heart Valve Diseases / surgery | 1 |  |
| Heart Valves / surgery | 1 |  |
| Hemoglobins / metabolism | 1 |  |
| Hepatectomy | 1 |  |
| Hepatectomy / methods | 1 |  |
| Hepatectomy / mortality | 1 |  |
| Hepatectomy / statistics & numerical data | 1 |  |
| Hospitalization | 1 |  |
| Hospitalization / statistics & numerical data | 1 |  |
| Hospitals / standards | 1 |  |
| Hospitals, Low-Volume | 1 |  |
| Insurance, Health / economics | 1 |  |
| Intensive Care Units / statistics & numerical data | 1 |  |
| Italy | 1 |  |
| Laparoscopy / adverse effects | 1 |  |
| Laparoscopy / methods | 1 |  |
| Length of Stay / statistics & numerical data | 1 |  |
| Liver | 1 |  |
| Logistic Models | 1 |  |
| Lung Diseases / mortality | 1 |  |
| Maternal Mortality | 1 |  |
| Medicaid / economics | 1 |  |
| Michigan | 1 |  |
| Morbidity / trends | 1 |  |
| Netherlands / epidemiology | 1 |  |
| New Zealand / epidemiology | 1 |  |
| Outcome Assessment, Health Care | 1 |  |
| Pancreatectomy / adverse effects | 1 |  |
| Pancreatic Diseases / surgery | 1 |  |
| Pancreatic Fistula / etiology | 1 |  |
| Pancreaticoduodenectomy / adverse effects | 1 |  |
| Pancreaticoduodenectomy / methods | 1 |  |
| Patient Acceptance of Health Care / statistics & numerical data | 1 |  |
| Patient Protection and Affordable Care Act | 1 |  |
| Patient Safety | 1 |  |
| Patient Safety / statistics & numerical data | 1 |  |
| Patient Selection | 1 |  |
| Pennsylvania / epidemiology | 1 |  |
| Peritoneal Neoplasms / epidemiology | 1 |  |
| Peritoneal Neoplasms / surgery | 1 |  |
| Personnel Staffing and Scheduling / organization & administration | 1 |  |
| Personnel Staffing and Scheduling / statistics & numerical data | 1 |  |
| Personnel, Hospital / supply & distribution | 1 |  |
| Postoperative Hemorrhage / etiology | 1 |  |
| Postoperative Hemorrhage / therapy | 1 |  |
| Practice Patterns, Physicians' / trends | 1 |  |
| Pregnancy | 1 |  |
| Pregnancy Complications / mortality | 1 |  |
| Preoperative Care | 1 |  |
| Proctectomy / adverse effects | 1 |  |
| Proctectomy / methods | 1 |  |
| Quality Improvement / statistics & numerical data | 1 |  |
| Quality Indicators, Health Care / statistics & numerical data | 1 |  |
| Quality of Health Care | 1 |  |
| Rectal Neoplasms | 1 |  |
| Rectum / surgery | 1 |  |
| Rehabilitation Centers / economics | 1 |  |
| Related informatio | 1 |  |
| Relief Work | 1 |  |
| Reoperation | 1 |  |
| Reoperation / statistics & numerical data | 1 |  |
| Reproducibility of Results | 1 |  |
| Risk Assessment | 1 |  |
| Safety-net Providers / statistics & numerical data | 1 |  |
| Sepsis | 1 |  |
| Spain / epidemiology | 1 |  |
| Stomach Neoplasms / complications | 1 |  |
| Stomach Neoplasms / pathology | 1 |  |
| Stomach Neoplasms / surgery | 1 |  |
| Surgical Procedures, Operative | 1 |  |
| Surgical Procedures, Operative / adverse effects | 1 |  |
| Surgical Procedures, Operative / mortality | 1 |  |
| Surgical Procedures, Operative / statistics & numerical data | 1 |  |
| Survival Rate | 1 |  |
| Travel | 1 |  |
| Treatment Failure | 1 |  |
| Wounds and Injuries / economics | 1 |  |
| Wounds and Injuries / rehabilitation | 1 |  |

**Supplementary Figure 1. Words cloud of MeSh terms**


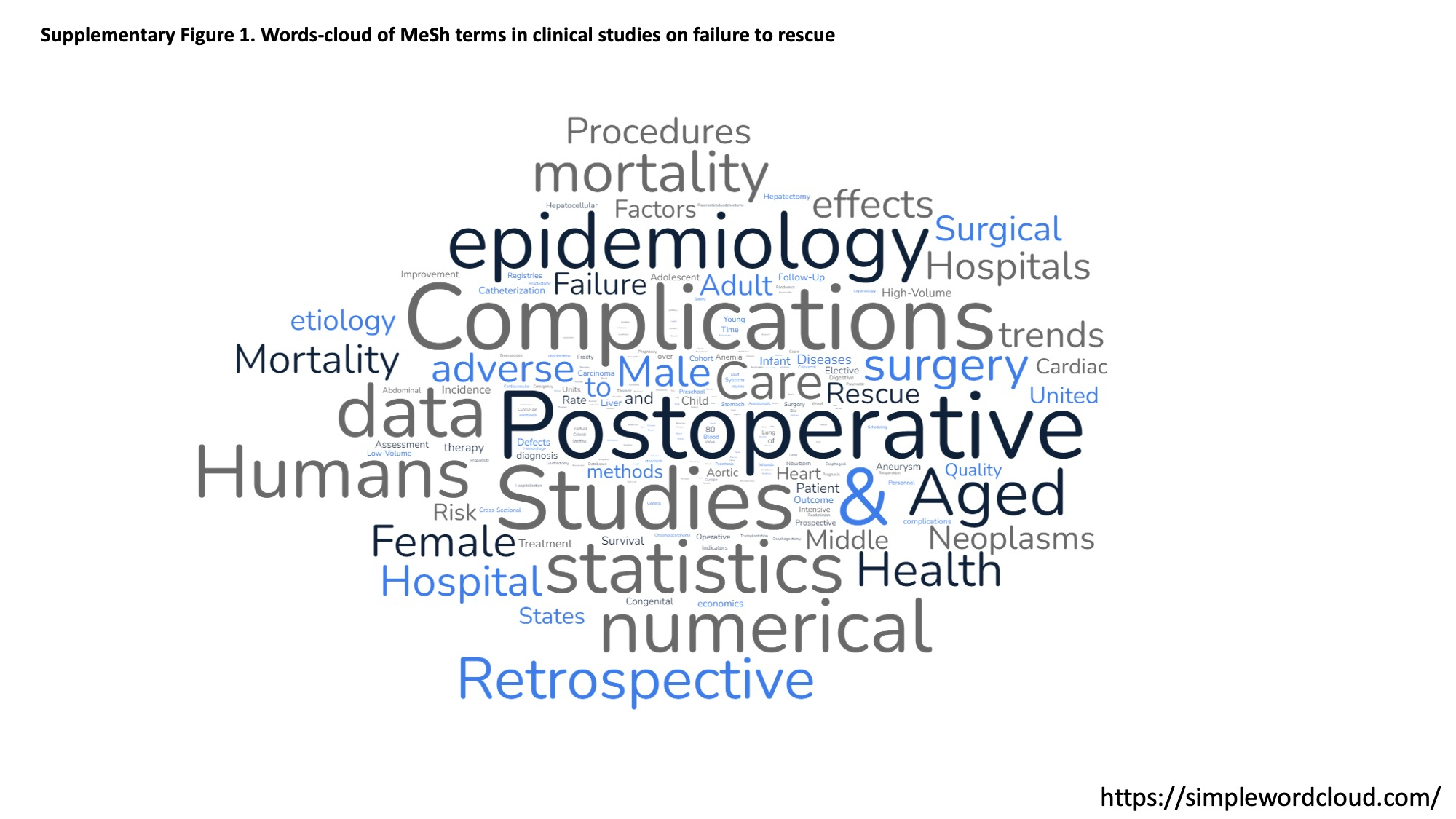


**Supplementary References - Manuscripts included in the analysis**

| Short Details | Title | Identifiers |
| --- | --- | --- |
| Hatchimonji JS. 2020 | A 'weekend effect' in operative emergency general surgery | PMID:31744597 |
| Osorio J. 2021 | Analysis of outcomes of emergency general and gastrointestinal surgery during the COVID-19 pandemic | PMID:34535796 |
| D'Oria M. 2021 | Association Between Hospital Volume and Failure to Rescue After Open or Endovascular Repair of Intact Abdominal Aortic Aneurysms in the VASCUNET and International Consortium of Vascular Registries | PMID:34225297 |
| Zogg CK. 2019 | Association of Medicaid Expansion With Access to Rehabilitative Care in Adult Trauma Patients | PMID:30601888 \| PMCID:PMC6537775 |
| Sánchez-Velázquez P. 2019 | Benchmarks in Pancreatic Surgery: A Novel Tool for Unbiased Outcome Comparisons | PMID:30829701 |
| Hatchimonji JS. 2021 | Differences Between Center-level Outcomes in Emergency and Elective General Surgery | PMID:33387728 |
| Hervás MS. 2021 | Evaluation of the relationship between lactacidemia and postoperative complications after surgery for peritoneal carcinomatosis | PMID:32434292 \| PMCID:PMC7862932 |
| Rosero EB. 2020 | Failure to rescue after major abdominal surgery: The role of hospital safety net burden | PMID:32199603 |
| Grönroos-Korhonen MT. 2022 | Failure to rescue after reoperation for major complications of elective and emergency colorectal surgery: A population-based multicenter cohort study | PMID:35927079 |
| O'Byrne ML. 2019 | Failure to Rescue as an Outcome Metric for Pediatric and Congenital Cardiac Catheterization Laboratory Programs: Analysis of Data From the IMPACT Registry | PMID:31619106 \| PMCID:PMC6898805 |
| Osho AA. 2020 | Failure to Rescue Contributes to Center-Level Differences in Mortality After Lung Transplantation | PMID:31470009 |
| Minor S. 2022 | Failure to rescue in emergency general surgery in Canada | PMID:35318241 \| PMCID:PMC9259385 |
| Wallen TJ. 2020 | Failure to Rescue in Humanitarian Congenital Cardiac Surgery | PMID:31610169 |
| Osho AA. 2020 | Failure to rescue in the era of the lung allocation score: The impact of center volume | PMID:31982094 |
| Francica A. 2022 | Gender-related presentation, surgical treatment, outcome and failure to rescue after surgery for type A aortic dissection: results from a multicentre registry | PMID:35640136 |
| van Groningen JT. 2020 | Identifying best performing hospitals in colorectal cancer care, is it possible? | PMID:32178963 |
| Scali ST. 2020 | Impact of hospital volume on patient safety indicators and failure to rescue following open aortic aneurysm repair | PMID:31515178 |
| Osorio J. 2021 | Improved postoperative outcomes and reduced transfusion rates after implementation of a Patient Blood Management program in gastric cancer surgery | PMID:33267997 |
| Duclos C. 2024 | Management and outcomes of hemorrhage after distal pancreatectomy: a multicenter study at high volume centers | PMID:37951805 |
| Filmann N. 2019 | Mortality after liver surgery in Germany | PMID:31339558 |
| LiverGroup.org Collaborative*. 2023 | Outcomes of elective liver surgery worldwide: a global, prospective, multicenter, cross-sectional study | PMID:38258997 \| PMCID:PMC10720814 |
| Thibault D. 2019 | Postoperative Transcatheter Interventions in Children Undergoing Congenital Heart Surgery | PMID:31159564 \| PMCID:PMC6705420 |
| Ubels S. 2023 | Practice variation in anastomotic leak after esophagectomy: Unravelling differences in failure to rescue | PMID:36732207 |
| Ardito F. 2020 | The Impact of Hospital Volume on Failure to Rescue after Liver Resection for Hepatocellular Carcinoma: Analysis from the HE.RC.O.LE.S. Italian Registry | PMID:32889868 |
| Dewan KC. 2021 | The Role of Frailty in Failure to Rescue After Cardiovascular Surgery | PMID:32866481 |
| Hawkins RB. 2019 | Travel distance and regional access to cardiac valve surgery | PMID:31374597 \| PMCID:PMC6776674 |
| Abdelsattar ZM. 2020 | Understanding Failure to Rescue After Esophagectomy in the United States | PMID:31706867 |
| Osorio J. 2022 | Use of failure-to-rescue after emergency surgery as a dynamic indicator of hospital resilience during the COVID-19 pandemic. A multicenter retrospective propensity score-matched cohort study | PMID:36089261 \| PMCID:PMC9458615 |
| Sheetz KH. 2019 | Variation in Surgical Outcomes Across Networks of the Highest-Rated US Hospitals | PMID:30865220 \| PMCID:PMC6583390 |
| Spence RT. 2021 | Will My Patient Survive an Anastomotic Leak? Predicting Failure to Rescue Using the Modified Frailty Index | PMID:33098049 |
|  | Interhospital failure to rescue after coronary artery bypass grafting | PMID:33712236 |
| Int J Qual Health Care. 2022 Nov 17 | Abdominal surgical trajectories associated with failure to rescue. A nationwide analysis | PMID:36287078 |
| J Surg Res. 2019 Mar | The Location and Timing of Failure-to-Rescue Events Across a Statewide Trauma System | PMID:30691839 |
| JAMA Surg. 2021 Jun 1 | Decreasing Failure-to-Rescue From Severe Maternal Morbidity at Cesarean Delivery: Recent US Trends | PMID:33881490 |
| BMJ Open. 2023 Nov 17 | Hospital variations in failure to rescue after abdominal surgery: a nationwide, retrospective observational study | PMID:37977874 |
| Surg Endosc. 2022 Jan | Failure to rescue following proctectomy for rectal cancer: the additional benefit of laparoscopic approach in a nationwide observational study of 44,536 patients | PMID:33871717 |
| Ann Surg. 2019 Jul | Association Between Hospital Staffing Models and Failure to Rescue | PMID:29557884 |
| Int J Qual Health Care. 2021 Mar 11 | Differences in organization of care are associated with mortality, severe complication and failure to rescue in emergency colon cancer surgery | PMID:33677517 |
